# Orthostatic hypotension and subjective symptomatic orthostasis in Parkinson’s disease: Associations and correlations

**DOI:** 10.1016/j.prdoa.2024.100262

**Published:** 2024-07-09

**Authors:** Jillian M. Heisler, Jon Toledo-Atucha, Chih-Chun Lin, Harsh N. Patel, William G. Ondo

**Affiliations:** a Work done while at Stanley H. Appel Department of Neurology, Houston Methodist Hospital, Houston, TX, USA; b Department of Neurology, Columbia University Irving Medical Center, New York, NY, USA; c Weill Cornell Medical School, Dept of Neurology, USA

**Keywords:** Parkinson’s disease, Orthostatic hypotension, Orthostasis, Blood pressure, Dopaminergics

## Abstract

**Background:**

Both measured orthostatic hypotension and symptomatic orthostasis are common in PD but their relationship is unclear.

**Objective:**

We aim to determine clinical predictors of both measured orthostatic hypotension and reported symptomatic orthostasis in PD, including the impact of “on”/“off” status and seasons, and to determine the correlation between measured OH and subjective orthostasis.

**Methods:**

We analyzed BP readings, demographic and disease state predictors for both 1. Measured blood pressure OH criteria and 2. The subjective report of orthostatic symptoms, using logistic regression analyses from an initial “on” motor state clinical visit in all PD patient visits. We then correlated subjective orthostasis symptoms with BP measurements. We also compared intra-subject BP measures in PD patients seen in both “on” and “off” states, and when seen “on” in both summer and winter.

**Results:**

723 consecutive visits over 2 years identified 250 unique PD individuals. Subjective orthostasis was reported by 44 % and “on” measured OH (>20 drop in SBP or 10 DBP upon standing) was seen in 30 %. Measured OH did not significantly correlate with any assessed clinical feature or specific medicine. Subjective orthostasis correlated most with older age, dementia, and L-dopa use. Subjective orthostasis correlated equally with absolute lower measured standing SBP and the drop in SBP from sitting to standing. Compared to the “off” state, “on” state showed lower sitting and standing SBP, more than DBP, but no significant change in BP drop upon standing. Seasons did not impact measured BP.

**Conclusions:**

Both OH and symptomatic orthostasis are common. Dopaminergic medications did not cause traditionally defined OH but lowered all SBP (sitting and standing) and thus reduced pulse pressure, possibly by increasing arteriole compliance simply by reducing motor tone, as this BP-lowering effect may be specific to Parkinsonism.

## 1 Introduction

Orthostatic hypotension (OH) is a common autonomic symptom, impacting 30–60 % of Parkinson’s disease (PD) patients [1]. Classic symptoms of OH include lightheadedness, dizziness, and syncope, but can also include generalized weakness, vision changes, fatigue, difficulty concentrating, and posterior headache. The classic definition of OH, a drop in systolic blood pressure (SBP) upon standing of >20 mmHg or diastolic blood pressure (DBP) drop >10 mmHg, may not capture all PD patients experiencing subjective orthostasis. It is unclear if the widely accepted definition of OH, or lower absolute systolic or diastolic standing blood pressure, or some other feature, most correlate with orthostatic symptoms and disability in PD [2].

The pathophysiology of OH in PD, and especially the mechanism by which L-dopa lowers BP in PD is incompletely understood. OH has been clinically associated with many different clinical features including older age, disease duration, dementia, and polypharmacy [3], [4], [5], [6], [7]. Tilt-table testing abnormalities in early PD also correlate with symmetric dopamine loss, especially in the anterior putamen in one dopamine transporter imaging study [8]. However, OH in untreated PD does not clearly correlate with cardinal motor signs [3], [4], [5], [6], [7].

We aim to determine factors that may associate with or contribute to measured OH, and symptomatic orthostasis, including the impact of seasons, “on”/“off” status, and whether the classic definition of OH or lower absolute standing SBP or DBP better correlate with symptomatic orthostasis in a large PD cohort.

## 2 Methods

Medical records of all consecutive PD patients seen in the Houston Methodist Movement Disorders Clinic between July 2017 and August 2019 were reviewed for demographics (sex, age, duration of PD) and other clinical features, including hallucinations, dementia, “on”/“off” status at that visit, time of year of that visit, anti-hypertensive medicines, and different PD medication classes. To avoid selection bias, we included all PD patients regardless of co-morbidities.

We gathered SBP and DBP in seated and standing positions (1 min apart) from the same calibrated automated sphygmomanometer. OH was defined as a drop of >20 mmHg in SBP or >10 mmHg in DBP. We analyzed demographics, and specific clinical features to assess associations with both measured blood pressure OH criteria, and the subjective report of orthostasis with logistic regression analyses, using only the first “on” motor state clinical visit, if there were multiple visits. Subsequent “on” visits and “off” visits were excluded from this statistic. “Off” visits were practically defined off from withholding any PD medication on the day of evaluation (at least 12 h). We also compared symptomatic orthostasis with the BP measurements to assess which aspect of BP (standing SBP, standing DBP, drop in SPB, drop in DBP) best correlated with subjective report of orthostasis. Variables were transformed as necessary to achieve a normal distribution. Power transformations were used to normalize the data distribution in parametric analyses.

Two separate analyses included additional data. We compared individuals with both an “on” and “off” medication state visit to evaluate the impact of the dopaminergic clinical state on BP measurements. We also evaluated patients who had both an “on” summer visit (May-Aug) and “on” winter visit (Nov-Feb) to see if the season impacted BP.

For univariate group comparisons (presence or absence of OH), we used t-tests for quantitative variables and chi-squared tests for categorical variables. For multivariable analyses, we used linear regression models. We used paired *t*-test and paired samples Wilcoxon test to compare the BP values in PD patients based on motor state (“off” vs. “on”) and season (summer vs. winter), based on the normal distribution of the data. We used false discovery rate correction to adjust for multiple comparisons. All analyses were performed using R-4.2.1.

## 3 Results

We evaluated 723 consecutive PD patient visits. Complete orthostatic BP measurements (sitting and standing) were not available in 122 visits due to: a documented inability to stand (n = 31), inability for the machine to obtain a standing BP (n = 2), refusal to perform standing BP (n = 3), or unspecified (n = 86). For the main cross-sectional analyses, we analyzed the data from the initial “on” visit for each subject, resulting in 250 different individual subjects (175 male, age 68.6 ± 8.7 years) with complete BP data (Table 1). For two separate analyses, 52 individuals had both an “on” and “off” visit to compare, and 90 individuals had both winter “on” and summer “on” visits to compare.

**Table 1:** Associations with measured “On” medicine orthostatic hypotension (SBP drop > 20 or DBP drop > 10).

	Total population [n = 250]	Measured OH YES n = 75 [30 %]	Measured OH NO n = 175 [70 %]	P value for change	Multicomp adjusted
Sex [Men]	175 [70 %]	57 [76 %]	118 [67.4 %]	0.23	0.54
Age [SD]	68.6 [8.7]	69.5 [7.7]	68.3 [9]	0.32	0.54
Dementia	36 [14.4 %]	13 [17.3 %]	23 [13.1 %]	0.52	0.75
Hallucinations	48 [19.2 %]	18 [24 %]	30 [17.1 %]	0.27	0.54
Any Rx for Hypertension	122 [48.8 %]	39 [52 %]	83 [47.4 %]	0.60	0.75
Amantadine	28 [11.2 %]	9 [12 %]	19 [10.9 %]	0.97	1.00
Dopamine agonists	110 [44 %]	27 [36 %]	83 [47.4 %]	0.13	0.54
MAO-B	117 [46.8 %]	35 [46.7 %]	82 [46.9 %]	1	1
L-dopa	216 [86.4 %]	72 [96 %]	144 [82.3 %]	0.007	0.07
Heart Rate Change	−3.3 [7.4]	−4.0 [8]	−2.9 [7.2]	0.30	0.54

OH, as assessed by the measured standard definition of OH (20 mmHg drop in SBP or 10 mmHg drop in DBP), was observed in 75/250 (30 %) of individuals during their initial “on” visit. Upon standing, there was a modest increase in heart rate (HR), mean 3.3 beats per minute (BPM), which did not differ in subjects with vs. without measured OH (Table 1). No assessed intrinsic clinical demographic or symptom (sex, age, dementia, hallucinations, hypertension as assessed by the use of anti-hypertensive medicines) associated with measured OH (Table 1). Measured OH was associated with L-dopa in univariant analysis only. There was no association with amantadine, dopamine agonists, MAO-B inhibitors, or medicines used to treat hypertension.

Subjective symptomatic orthostasis was reported by 109/247 (44 %) of participants, in three, the chart data was equivocal. Univariate clinical features that correlated with symptomatic orthostasis included older age, dementia, and L-dopa use; with multivariate testing, only L-dopa (used by 86 % of subjects) remained close to significant (p < 0.07) (Table 2).

**Table 2:** Associations with report of subjective orthostasis.

	Total population (n = 247)	Subjective OH YES, n = 109 (44 %)	Subjective OH NO, n = 138 (56 %)	P value Univariate	Multi-comp adjusted
Age [S.D.]	68.6 [8.7]	70 [7.6]	67.5 [9.4]	0.024	0.11
Female Sex	75 (30.0 %)	36 (33 %)	37 (26.8 %)	0.36	0.49
Dementia	36 (14.4 %)	22 (20.2 %)	14 (10.1 %)	0.047	0.14
Hallucinations	48 (19.2 %)	25 (22.9 %)	23 (16.7 %)	0.28	0.49
Any Rx for BP	122 (48.8 %)	59 (54.1 %)	63 (45.7 %)	0.23	0.49
Amantadine	28 (11.2 %)	11 (10.1 %)	17 (12.3 %)	0.73	0.73
Dopamine agonists	110 (44.0 %)	45 (41.3 %)	65 (47.1 %)	0.43	0.49
MAO-B	117 (46.8 %)	48 (44 %)	69 (50 %)	0.42	0.49
L-dopa	216 (86.4 %)	102 (93.6 %)	114 (82.6 %)	0.017	0.11

In a univariate analysis of “on” medicine blood pressure readings, the subjective report of orthostasis correlated about equally with absolute lower standing SBP, absolute lower standing DBP, drop in SBP (sitting to standing), and drop in DBP (Table 3). Sitting SBP, sitting DBP, pulse, and change in pulse from sitting to standing did not correlate with symptomatic orthostasis.

**Table 3:** Assessment of measured “on” medicine blood pressure measures to identify correlates with subjective orthostasis.

	Subjective orthostasis**-** yes n = 109	Subjective orthostasis**-** no n = 138	P value for change	Multi-comp adjusted
SBP standing	119.3 [23.8]	128.2 [20.6]	0.0005	0.0015
DBP standing	72.8 [12]	78 [10.8]	0.0003	0.0015
SBP sitting	132.9 [23.7]	135.7 [20.3]	0.23	0.35
DBP sitting	76.9 [10.3]	78.8 [8.9]	0.10	0.18
Drop in SBP	13.6 [14.1]	7.5 [13.7]	0.0007	0.0015
Drop in DBP	4.1 [7.7]	0.8 [7.1]	0.0005	0.0015
Heart Rate standing	79.7 [12.8]	79.3 [13]	0.75	0.75
Heart Rate sitting	76.5 [13.4]	75.8 [12.3]	0.75	0.75
Heart Rate Change	−2.9 [7]	−3.5 [7.9]	0.57	0.73

Intra-subject BP measurements comparing “off” vs. “on” visits (n = 52) showed the greatest difference in standing SBP (mean: 131.1 vs. 123.7, p < 0.05) and sitting SBP (mean: 143.9 vs. 135.4, p < 0.05) but less change in DBP readings (NS) (Table 4). Mean sitting to standing drop in SBP and DBP (defined OH) was not significantly different in the “off” vs. “on” state. HR and sit-to-stand change in HR were not impacted by “off”/“on” status. Therefore, the “on” state mostly lowered SBP equally in all conditions.

**Table 4:** Intra-subject comparison of “on” medicine vs. “off” medicine blood pressure readings.

	“Off” meds n = 52	“On” meds n = 52	P value for change	FDR
SBP standing	131.1 [20.6]	123.7 [18.9]	0.015	0.045
DBP standing	78.3 [9.9]	75.4 [10.1]	0.066	0.15
SBP sitting	143.9 [20.4]	135.4 [20.2]	0.015	0.045
DBP sitting	81.1 [8.7]	76.8 [8]	0.0069	0.045
Drop in SBP	12.9 [12.5]	11.7 [14.5]	0.60	0.91
Drop in DBP	2.8 [8]	1.4 [7.2]	0.31	0.56
Heart Rate standing	80.4 [16.4]	80.2 [13]	0.97	0.97
Heart Rate sitting	77.6 [13.9]	77.5 [13.8]	0.93	0.97
Heart Rate Change	−3.4 [10]	−2.7 [9.3]	0.76	0.97

Pulse pressure (SBP-DBP) was generally high (normally 40–60 mm Hg). The mean “on” sitting (n = 250) was 56.3 ± 16.8, with 35.2 % of “on” subjects were greater than 60 (abnormal). “Off” sitting pulse pressure (n = 52) was higher 62.9 ± 17.7, with 53.8 % of subjects greater than 60.

Intra-subject “on” BP measurements did not differ between summer and winter (n = 90) [Supple Table 1].

## 4 Discussion

In our PD population, both symptomatic orthostasis (44 %) and measured OH (30 %) were common. OH was a neurogenic pattern without compensatory HR elevation upon standing. “On” measured OH was only associated with L-dopa use, whereas subjective symptomatic orthostasis was associated with older age, dementia, and L-dopa. Subjective orthostasis correlated equally with both the drop in SBP upon standing and absolute lower standing SBP while standing. “On” state BP measurements demonstrated a greater reduction in SBP, more than DBP, both standing and sitting, but did not have more actual BP drop upon standing, so the “on” dopaminergic therapy state did not increase classically defined OH, but did lower standing SBP, and could cause symptomatic orthostasis. We did not find a significant effect of season (outside temperature) on measured OH, despite the fact that BP is lowered by increased ambient temperature (vasodilation and fluid loss) [9]**,** and the anecdotal impression that patients complain of orthostasis more during summer, potentially because indoor temperatures in the clinic are relatively homogeneous.

As expected, our measured OH was consistent with a “neurogenic OH”, as the pulse did not increase upon standing in those with a BP drop [10]. The mild pulse increase (3.2 BPM) was seen equally in those with or without BP drops, and is consistent or less than seen in a normal population. Neurogenic OH in PD is generally thought to result from postganglionic sympathetic efferent nerves innervating both the heart [11] and blood vessels. However, the relative contribution of each is not known [12], [13].

In our study, patients seen “off” dopaminergic medications had higher absolute SBP while both sitting and standing, compared to “on” medications. Pulse, and absolute drop in standing BP were largely unaffected by “on”/“off” status. Therefore, the main net difference was greater pulse pressure (SBP-DBP) in the “off” state. Pulse pressure is felt to be proportional to cardiac stroke volume and inversely proportional to arterial compliance. Since there is minimal evidence to suggest greater stroke volume in “off” state PD, this suggests that reduced arterial compliance, possibly simply due to general rigidity, could cause the increased pulse pressure and the relatively higher SBP seen in the “off” state, compared to the “on” state when the motor tone normalizes. This observation has potential implications. First, this may result in a diagnosis of hypertension if BP is measured while in the “off” state, supported by the fact that 44.4 % of our subjects took medications to lower BP despite many having OH. Second, the increased pulse pressure seen in the “off” state, but reduced in their “on” state, is strongly associated with cardiovascular risk, possibly more than overall hypertension, arguing that fluctuations could increase cardiovascular risk [14].

The mechanism of the vasodepressive effect [15] with L-dopa is debated and has been attributed to reduced plasm catecholamine levels, reduced cardiac stroke volume and output, and increased venous compliance [16]. The lack of greater standing BP drop in our study suggests dopaminergic medications do not alter sympathetic compensation (vasoconstriction), specifically when a person stands. Our data could argue that dopaminergic medications do not abnormally or directly lower BP at all, but rather “normalize” BP in PD to its abnormally low state by lowering pulse pressure (usually 40–60) via lessened motor tone, and that the “off” rigid state relatively increases SBP in all conditions. This is supported by the recent report that drop in standing BP following L-dopa challenge correlates with improvement in PD motor features [17], and that acute activation of sub-thalamic nucleus deep brain stimulation both improves motor function and lowers standing BP [18]. Furthermore, the lack of measured OH or symptomatic orthostasis in restless legs syndrome studies of dopaminergics, which did use smaller doses than when used in PD, and anecdotally when used clinically, often at much larger doses, suggest dopaminergic medications only lower SBP in Parkinsonism. This may also explain the disagreement in the PD literature where dopaminergic medications are not associated with classically measured OH [19], compared to clinical practice, and some studies that do show dopaminergic medications frequently cause subjective symptomatic orthostasis.

There is inconsistent correlation between measured OH and orthostasis [5]. Hellman *et al*. reported that orthostasis in male patients without measured OH were more likely to demonstrate abnormal BP measurements during 3-phase Valsalva maneuver than asymptomatic patients, in fact to a degree similar to those with measured OH, suggesting that the symptoms still related to BP [20]. Our data could not specifically associate OH more with an absolute lower standing BP versus a drop in sitting to standing BP. However, this argues that the lower absolute standing SBP is more culpable. Palma *et al* also felt that a lower standing mean arteriole pressure, compared to drop in BP, more correlated with symptomatic orthostasis [2].

Specific limitations of our study include the retrospective chart review data acquisition. For example, symptomatic orthostasis wasn’t based on specific clinical scales but rather on the clinical impression of the movement disorder specialist, as documented in the chart, which may result in under-reporting. Intra-subject assessments (on vs. off, and “winter” vs. “summer”) did not control all potential variables that may have differed, including variable like disease duration, changes in other medical conditions and both PD and other medications. BP measurements were done at 1 min, not 3 min which is used in some, but not all, formal trials. Although done mostly to expedite intake, 1-minute OH correlates more with orthostatic symptoms, including falls, compared to later OH assessments in the general geriatric population, so it may have more utility [21]. We also measured sitting, rather than supine BP, which may have lessened the incidence of measured OH. Most patients who did not receive orthostatic BP measurements had difficulty standing, including many in whom this was “not documented”, which may have lessened data acquisition for the most severe patients, although this should not have biased the existing data. To avoid selection bias in a “real world” cohort, we did not exclude any available subject, even if they had other possible explanations for OH, so this could result in additional unassessed variables. Nevertheless, we think this large population analysis offers several unique observations and a novel hypothesis regarding BP drop associated with L-dopa.

## Author roles

Jillian M. Heisler: 1B, 1C, 3B.

Jon Toledo-Atucha: 2A, 2B, 2C, 3B.

Chih-Chun Lin: 1B, 1C, 3B.

Harsh N. Patel: 1B, 1C, 3B.

William G. Ondo: 1A, 1B, 1C, 2A, 2C, 3A.

## Disclosures regarding work

No specific funding was received for this work, and the authors declare that there are no conflicts of interest relevant to this work.

## Financial disclosures past 12 months

Jillian M. Heisler: none.

Jon Toledo-Atucha: none.

Chih-Chun Lin: Research grants from the National Ataxia Foundation.

Harsh N. Patel: none.

William G. Ondo: Research grants from Biogen, Sun, Restless Legs Syndrome Foundation, Parkinson’s Study Group, Dystonia Coalition (NIH), Cerevel, SCION, and Harmony. Honorarium for speaking bureau from: TEVA, ACADIA, Acorda, Neurocrine, Supernus, Allergan, and Kyowa Kirin. Consulting fees from Merz, Sage, Jazz, XWPharma, Neurocrine, Supernus, Amneal. Royalties from the books *Movement Disorders in Psychiatry, and UpToDate*.

## Ethical compliance

The protocol was reviewed and approved by the Houston Methodist Research Institute IRB.

We confirm that we have read the Journal’s position on issues involved in ethical publication and affirm that this work is consistent with those guidelines.

## CRediT authorship contribution statement

**Jillian M. Heisler:** Writing – review & editing, Data curation. **Jon Toledo-Atucha:** Writing – review & editing, Formal analysis, Data curation. **Chih-Chun Lin:** Writing – review & editing, Data curation. **Harsh N. Patel:** Writing – review & editing, Data curation. **William G. Ondo:** Writing – original draft, Project administration, Methodology, Formal analysis, Conceptualization.

## Declaration of competing interest

The authors declare that they have no known competing financial interests or personal relationships that could have appeared to influence the work reported in this paper.
