# Supplementary material for: Orthostatic hypotension and subjective symptomatic orthostasis in Parkinson’s disease: Associations and correlations
Source: Clin Park Relat Disord. 2024 Jul 9;11:100262. doi: 10.1016/j.prdoa.2024.100262 (PMC11298931; doi:10.1016/j.prdoa.2024.100262)
Supplement: Supplementary Data 1 [file mmc1.docx]

**Supple Table 1: Intra-subject comparison of “on” medicine summer vs. winter blood pressure readings**

|  | Summer months  (n=90) | Winter months  (n=90) | P value for change | Multi-comp adjusted |
| --- | --- | --- | --- | --- |
| SBP standing | 125 [21.7] | 126.6 [23.4] | 0.51 | 0.94 |
| DBP standing | 75.4 [9.1] | 76.8 [10.4] | 0.26 | 0.94 |
| SBP sitting | 135.7 [21.5] | 138.1 [23.4] | 0.88 | 0.94 |
| DBP sitting | 77.7 [9.8] | 78.8 [9.9] | 0.65 | 0.94 |
| Drop in SBP | 10.7 [15.3] | 11.5 [12.1] | 0.55 | 0.94 |
| Drop in DBP | 2.3 [6.7] | 2 [7.9] | 0.80 | 0.94 |
| Heart Rate standing | 78.6 [12.5] | 77.4 [12.1] | 0.45 | 0.94 |
| Heart Rate sitting | 75.4 [12.5] | 74.7 [13] | 0.12 | 0.94 |
| Heart Rate change | -3.3 [7.8] | -2.9 [8.7] | 0.94 | 0.94 |
